# Managing Patient Factors in the Assessment of Swallowing via Telerehabilitation

**DOI:** 10.1155/2012/132719

**Published:** 2012-09-13

**Authors:** Elizabeth C. Ward, Shobha Sharma, Clare Burns, Deborah Theodoros, Trevor Russell

**Affiliations:** ^1^School of Health & Rehabilitation Sciences, The University of Queensland, St. Lucia, Brisbane, QLD 4072, Australia; ^2^Centre for Functioning and Health Research, Queensland Health, Brisbane, QLD 4102, Australia; ^3^School of Rehabilitation Sciences, National University of Malaysia, Bangi Selangor 43600, Malaysia; ^4^Speech Pathology Department, Royal Brisbane & Women's Hospital, Herston, Brisbane, QLD 4029, Australia

## Abstract

Undoubtedly, the identification of patient suitability for a telerehabilitation assessment should be carried out on a case-by-case basis. However, at present there is minimal discussion of how telerehabilitation systems can accommodate and adapt to various patient factors, which may pose challenges to successful service delivery. The current study examines a subgroup of 10 patients who underwent an online assessment of their swallowing difficulties. Although all assessments were completed successfully; there were certain patient factors, which complicated the delivery of the online assessment session. The paper presents a discussion of the main patient factors observed in this cohort including the presence of speech and/or voice disorders, hearing impairment, dyskinesia, and behavioural and/or emotional issues and examines how the assessment session, the telerehabilitation system, and the staff involved were manipulated to accommodate these patient factors. In order for telerehabilitation systems to be more widely incorporated into routine clinical care, systems need to have the flexibility and design capabilities to adjust and accommodate for patients with varying levels of function and physical and psychological comorbidities.

## 1. Introduction

Telerehabilitation services often involve intensive, detail-oriented, and interactive assessments. Hence it is accepted that patients are ideally served by systems and technology designed to optimise high quality visual and audio during a real-time interaction [1]. It is, however, equally important that any telerehabilitation system or service is designed to be sensitive to and accommodate the needs of the end user [2, 3]. Optimising both the equipment technology and the adaptability/usability of the system helps to ensure the development of systems and services that function well and are sufficiently flexible to adapt to patients with various levels of capability. 

The use of telerehabilitation to conduct clinical assessments of dysphagia is an area of practice which is currently still in its infancy. Hence the system requirements necessary to optimise capabilities for both the user and the patient during online assessments of swallowing are still being established. Initial work by Lalor et al. [4] described the use of a videoconferencing system with fixed cameras to conduct an assessment of swallowing and language for a remote patient with global aphasia and severe dysphagia. Although session objectives were able to be met, the researchers reported facing multiple difficulties during the assessment, which were attributed to patient factors, equipment limitations, and technical (visual, audio) aspects of the session. Hence improvements in system technology and design were required. It was also clear that having staff available at the patient end during the assessment was necessary.

Building on these lessons learnt, Sharma et al. [5] published a description of purpose-built telerehabilitation system for conducting clinical assessments of patients with dysphagia. This system ran off two notebook computers equipped with custom video conferencing software for real-time videoconferencing. The system at the patient end was controlled by the online clinician and required no intervention from the patient. Fixed and free standing cameras with zoom capabilities as well as a free field and a lapel microphone were incorporated to enhance the visual and auditory information. Store and forward capability was available to enable the session to be recorded and a split screen display allowed the patient to see themselves (for visual feedback during oromotor tasks) as well as the online clinician during the sessions. Adjustments were made to the assessment process, including use of a laryngeal marker and clear utensils to help enhance information provided to online clinician. A patient's assistant was also incorporated at the patient end to assist with the manual tasks of food/fluid trials. The pilot trial of this system with 10 standardised patients revealed its potential to provide necessary information for diagnostic decisions about swallow safety. However, the authors noted that testing with a true patient cohort was necessary to determine the true functionality of the system [5]. 

Ward et al. [6] subsequently used the same system in a cohort study of 40 dysphagic patients. That study found that the level of agreement between the clinical decision made by the online clinician was comparable to those made by a face-to-face (FTF) clinician who simultaneously assessed the patients. Furthermore, the online clinician expressed high satisfaction with the service and found that the equipment provided good audio and visual quality and was easy to use for most of the sessions. There were, however, a small proportion of patients though for whom the online clinician felt a traditional assessment may have been more appropriate. This data, which appeared to pertain to patient factors, was not elaborated on any further in the paper. 

As part of the development and evaluation of any new telerehabilitation application, there needs to be systematic investigation of the system design elements and their functionality when applied with the desired population or rehabilitation service. Hence it is the aim of the current investigation to examine further the issues which potentially impacted on the service delivery of clinical dysphagia assessment via the telerehabilitation system as used by Ward et al. [6]. Recent guidelines note that “…the candidacy and appropriateness for telerehabilitation should be determined on a case by case basis with selections firmly based on clinical judgement, client's informed choice and professional standards of care” [7, page 664]. However, in order to make informed decisions regarding patient suitability for a particular type of telerehabilitation service, it is important that patient factors which challenge that system or service are known and considered, so that strategies that can facilitate successful online service delivery can be implemented.

## 2. Methods

Participants were selected from the original cohort of 40 dysphagic participants who participated in the study reported by Ward et al. [6]. Participant inclusion and exclusion factors for this total cohort are reported in detail in the Ward et al. [6] manuscript; however, in brief the 40 patients presented with mild to severe dysphagia from various aetiologies. These 40 participants then underwent an online clinical assessment of swallowing using the telerehabilitation system as detailed in Ward et al. [6]. All sessions were led by the online clinician, but both the online and an FTF clinician simultaneously assessed all patients. Before each assessment, the two clinicians were randomly assigned to be either the online clinician or the FTF clinician, hence each assessed 20 participants in the online role. Following each assessment, only the online clinician completed a questionnaire about the session [6]. Data collected from this questionnaire was reviewed and any participant who received either a rating of 3, indicating the clinician was unsure, or a rating of 1 or 2 indicating the clinician disagreed with the statement “I feel that the telerehabilitation system would be a more efficient means of service delivery for this particular patient,” was subsequently selected for inclusion in the current study. Ten of the 40 participants met this criteria; six received a rating of 3 (Participants no. 3, 6, 7, 8, 9, 10) and four received a rating of 2 (Participants no. 1, 2, 4, 5). Mean age of the 10 participants was 69 years (range 50 to 93 years) with four males and six females. Individual case details and dysphagia severity can be found in Table 1.

Notes made by the online clinician about the sessions for these 10 participants were subsequently reviewed, and common issues were noted. Specifically, four main participant-related issues were encountered, which were found to influence the clinicians' ratings of the efficiency of online assessment for each individual. The issues included the presence of (1) speech and/or voice disorders (six participants), (2) hearing impairments (three participants), (3) coexisting movement disorders (dyskinesia) (one participant), and (4) behavioural and or emotional issues (three participants) (Table 1). Some individuals (*n* = 3) had more than one issue impacting the session. 

However, despite the online clinician indicating that the telerehabilitation environment may not have been the most efficient means to assess these particular 10 individuals, a review of each session revealed that all assessments were still completed successfully, and a diagnostic decision was achieved for each individual. Furthermore, the level of agreement between the online and the FTF clinicians ratings for the primary diagnostic decisions relating to (a) safe fluid consistency and (b) safe food consistency revealed 100% exact agreement (Table 2). Hence in spite of the challenges certain patient factors created to conducting an assessment in the online environment, a comparable clinical diagnostic decision to that obtained FTF was still able to be achieved.

## 3. Results and Discussion

 The following sections discuss the four key patient factors, which were found to influence the telerehabilitation session and the strategies used to compensate for and adapt to these issues. 

### 3.1. Impact and Management of Speech and Voice Disorders

A weak and dysphonic voice quality and reduced speech intelligibility of six individuals (see Table 1) added complexity to the assessment sessions. While these did not directly inhibit the online speech pathologists (O-SP) ability to assess swallowing, it was more challenging to understand the patient clearly during conversations. Weak voice quality also impacted on the ability to hear any subtle changes in voice quality after swallow, forcing the O-SP to rely more heavily on other signs of potential aspiration risk. To help compensate, the O-SP frequently adjusted the volume of the throat microphone and frequently required clarification from the Assistant. For some individuals, the session duration was slightly increased which allowed for some repetition of tasks to check performance and a greater interaction between the online clinician and Assistant. These simple adjustments allowed all the clinically relevant information to be obtained for the successful completion of the swallowing assessment.

In the study by Hill et al. [8], which assessed apraxia in eleven participants, issues with occasional communication breakdown due to the severity of motor speech impairment were also highlighted as a challenge in the sessions. It was reported that the researchers occasionally had to allow the participants to rely upon writing as their mode of communication to repair online communication breakdowns. However, as the system used by Hill et al. did not allow for the capture of clear written messages online, the authors reported that participants became frustrated during the assessment. In the current study, the presence of the Assistant at the patient end helped to minimise the conversational breakdown between the O-SP and the participant. By attending to the participant and repeating and/or clarifying what had been said to the O-SP as needed, the negative impact of the speech and voice deficits was reduced and frustrations were minimised. 

### 3.2. Impact and Management of Hearing Impairment

For three individuals, the presence of unaided hearing impairment created challenges during the assessment. The hearing impairments caused occasional communication breakdowns between the participants and the O-SP. In all instances it is very probable that the telerehabilitation session would have run more smoothly if the hearing aids were available and in working order for the session. The experience with these three individuals highlights the need to ensure patients are adequately prepared prior to the session and clear instructions given to family/care staff to ensure patients who require hearing aids come adequately prepared for the session. 

Although the individuals were unaided during the session, it was possible to compensate for this and successfully complete the assessment. The Assistant helped by adjusting the speaker volume at the patient end, and by repeating the instructions of the online clinician directly to the patient. The O-SP also modified instructions to make them as short, simple, and clear as possible for the patient. Although it was not necessary for any of these three participants, preparation of simple written instructions for patients, which could be typed in by the online clinician and electronically posted onto the screen of the computer system at the patient's end, could also be used to help compensate for auditory-perceptual breakdowns.

Although the presence of a hearing impairment does not prohibit individuals from participating in telehealth services [9–12], in the current study the ability to manipulate the audio and visual signal was necessary to assist interactions online. Furthermore, the system allowed for clear vision of the online clinician, which helped the individuals with a hearing impairment gather information that was missed and interpret information provided through facial cues and gestures. Issues with occasional audio delays and unnatural eye contact (created by looking at the screen rather than into the eye of the web camera), however, were other issues that were noted to impact on exchanges and could be improved in the future.

### 3.3. Impact and Management of the Presence of Movement Disorders (Dyskinesia)

One patient presented with a diagnosis of Parkinson's disease and associated cervical dyskinesia. Due to her dyskinesia, she was unable to retain a position that maintained her within the usual visual field set for the web camera during assessments. Zoom capabilities were also of limited use. To compensate, the free standing camera, positioned for a wider angle field of view, was used to enable visualization of the head, neck, and upper torso. While not ideal, particularly during food and fluid trials when close focus on the mouth and upper throat is preferable, the wider angle helped compensate for the movement and maintain vision of the participant most of the time. The free standing camera could also be positioned by the Assistant relative to the participant, rather than constantly repositioning. 

A further issue with this participant was that she displayed signs of fatigue, and short breaks were needed throughout the session. The need for these short breaks lengthened the duration of the assessment session. The findings highlight the need to schedule a longer assessment session for certain individuals with more complex conditions.

### 3.4. Impact and Management of Behavioural/Emotional Issues

 Behavioural or emotional issues impacted the duration and the flow of three of the assessment sessions. For these individuals, the Assistant was instrumental in facilitating the sessions success. Previous studies have reported that attention deficits can be challenging to manage in an online environment [1, 13, 14]. However, having the Assistant present to refocus attention and minimise distractions greatly assisted the online clinical to manage and assess these individuals. The Assistants presence was also beneficial from the participant's perspective, as she was on hand to provide reassurance and emotional support to participants when needed.

Patient impulsivity raised challenges during the food/fluid trials. This was particularly an issue when participants took large or multiple sips of fluids during the trials, unheeding instructions from the O-SP. Again, in these cases, intervention by the Assistant helped to control the rate of oral intake and discourage talking until it was clear they had completed the swallow. One participant (Pt 10), who was largely noncommunicative, presented with a flat affect and provided delayed responses, was challenging to assess online. Strategies that helped during the session were to provide instructions slowly and for Assistant to repeat instructions to ensure that the participant attended to the tasks required.

For Participant 1, her emotional state impacted the flow of the session. Her acute awareness of her loss of function since surgery meant she frequently became emotional when she was unable to carry out tasks (e.g., in oromotor examination). She frequently required some time to regain composure to continue the assessment session. Reassurances from the O-SP and the physical presence of the Assistant helped to assist.

## 4. Conclusion

Dysphagia is a symptom caused by a wide range of aetiologies including neurological injury/insult, degenerative disorders, trauma, surgical interventions, and even the natural aging process. Hence there is a high likelihood that patients referred for a dysphagia assessment via telerehabilitation will present with a range of comorbidities as well as altered cognitive and emotional states. The current study demonstrates that in spite of the range of challenges raised by the altered capabilities of these 10 participants, all assessments were completed successfully through modifications of the current equipment and through the help of the Assistant at the patient end. It is acknowledged, however, that this is a small cohort and the systems capabilities to accommodate all possible patient factors cannot be considered to be complete. As new systems are proposed and technology advances, ongoing investigation into how these systems perform and can adjust to compensate for various patient factors will emerge. From such research, it will be possible in the future to more clearly identify the minimum telerehabilitation system requirements needed to assess patients with various comorbidities.

## Figures and Tables

**Table 1:** Description of presenting characteristics of the 10 participants and the key issues complicating the assessment session.

Pt.	Age (years)	Gender	Diagnosis	Dysphagia severity	Complex characteristics	Key issue/s
1	50	F	T2N1 SCC left tongue treated with left supramyohyoid dissection, resection (left) tongue, (left) posterior tongue and tonsil removed, wrap around (right) anterior tongue flap. Postoperative radiotherapy.	Moderate	Mild dysarthric speech, moderate-severe dysphonia (husky voice, reduced intensity), emotional psychosocial changes coping with acute changes to voice posttreatment as participant was a professional voice user.	Voice/Speech and Behaviour/Emotion
2	89	F	Hurthle cell thyroid cancer (widely invasive with recurrent laryngeal nerve palsy and subglottic stenosis)—treated with hemithyroidectomy and laser excision of stenosis/obstructive lesion. Postoperative radiotherapy.	Moderate severe	Severe dysphonia (hoarseness and breathiness).	Voice/Speech
3	59	M	T1N1 SCC of right lateral tongue managed with a right hemiglossectomy and right neck dissection (level 1–3) and postoperative chemoradiotherapy.	Moderate	Mild-moderate hearing loss, mild dysarthria	Hearing impairment
4	89	F	Olivopontine atrophy	Mild-Moderate	Moderate hearing loss, severe dysphonia (hoarseness)	Hearing impairment
5	69	M	Prior history of a T2N2c SCC of left base of tongue managed via chemoradiotherapy. Recently managed for osteoradionecrosis of right jaw, which was treated surgically with partial mandibulectomy and a fibular free flap.	Severe	Mild-moderate hypernasality with moderate-severe dysarthria	Voice/Speech
6	56	F	T4 N2 SCC of the left oropharynx, managed with chemoradiotherapy.	Mild-moderate	Mild reduction in attention span, easily distracted, self-conscious on web-camera, inappropriate timing of conversation.	Behaviour/Emotion
7	35	F	T4N0 SCC of the left tongue. Treated via a left hemiglossectomy with buccinators flap repair and left neck dissection (level 1–3) and adjuvant radiotherapy. Diffuse scleroderma post radiotherapy.	Severe	Moderate-severe dysarthria, clenching of teeth during speech production resulting in reduced intelligibility, mild hypernasality.	Voice/Speech
8	68	F	Parkinson's disease with cervical dyskinesia	Moderate	Uncontrolled head and neck movements, vocal tremors, severe generalised tremors.	Movement disorder and voice/speech
9	93	M	Prior history of Achalasia, CVA (no residual deficits), vascular dementia, Depression, lumbar spinal stenosis. At time of assessment was admitted with chest pain and vomiting and acopia.	Mild-Moderate	Reduced attention/engagement	Behaviour/Emotion
10	82	M	T3N2 SCC of oropharynx. Assessment conducted presurgery (planned intervention: total laryngectomy and bilateral neck dissection).	Moderate severe	Severe dysphonia (rough and hoarse voice, reduced intensity, occasional diplophonia), moderate hearing impairment.	Voice/Speech, and Hearing impairment.

Pt.: participant; M: male; F: female; CVA: cerebrovascular accident; SCC: squamous cell carcinoma; T: tumour size; N: nodal disease.

**Table 2:** Clinical decisions on safe food and food and fluid consistencies for each patient as made during simultaneous assessment by the online and face to face clinician.

Patient no.	Final food decision		Final fluid decision	
	Online	FTF	Online	FTF
1	Puree	Puree	Thin	Thin
2	Minced and Moist	Minced and Moist	Moderately thick	Moderately thick
3	Minced and Moist	Minced and Moist	Thin	Thin
4	Soft	Soft	Thin	Thin
5	Nil by mouth	Nil by mouth	Nil by mouth	Nil by mouth
6	Minced and Moist	Minced and Moist	Thin	Thin
7	Nil by mouth	Nil by mouth	Nil by mouth	Nil by mouth
8	Puree	Puree	Mildly thick	Mildly thick
9	Soft	Soft	Thin	Thin
10	Puree	Puree	Extremely thick	Extremely thick
